# Association of combined pulmonary fibrosis and emphysema in rheumatoid arthritis with high titer of rheumatoid factor and autoimmunity to the lung

**DOI:** 10.3389/fimmu.2025.1514552

**Published:** 2025-02-05

**Authors:** Aiping Ma, Renliang Huang, Jiaxi Guo, Guangdong Wang, Heqing Huang, Xinze Li, Shan Zhong, Yan Huang, Shaowei Lin, Yikai Lin, Qiaomiao Zhou, Susanne Krauss-Etschmann, Frank Petersen, Zhanxiang Wang, Xinhua Yu

**Affiliations:** ^1^ Department of Respiratory and Critical Medicine, The First Affiliated Hospital of Xiamen University, School of Medicine, Xiamen University, Xiamen, China; ^2^ Department of Genetics and Prenatal Diagnosis, Hainan Women and Children’s Medical Center, Haikou, Hainan, China; ^3^ Department of Rheumatology, The First Affiliated Hospital of Xiamen University, School of Medicine, Xiamen University, Xiamen, China; ^4^ NHC Key Laboratory of Tropical Disease Control, School of Tropical Medicine, Hainan Medical University, Haikou, Hainan, China; ^5^ Department of Pathology, The First Affiliated Hospital, School of Medicine, Xiamen University, Xiamen, China; ^6^ Department of Nuclear Medicine, The First Affiliated Hospital of Xiamen University, School of Medicine, Xiamen University, Xiamen, China; ^7^ Department of Radiology, The First Affiliated Hospital of Xiamen University, School of Medicine, Xiamen University, Xiamen, China; ^8^ Priority Area Chronic Lung Diseases, Research Center Borstel, Borstel, Germany; ^9^ Department of Neurosurgery, The First Affiliated Hospital of Xiamen University, Xiamen, China

**Keywords:** combined pulmonary fibrosis and emphysema (CPFE), connective tissue diseases (CTD), rheumatoid arthritis (RA), autoimmunity, rheumatoid factor (RF), human bronchial epithelial cells (HBEC), interstitial lung disease

## Abstract

**Background:**

Combined pulmonary fibrosis and emphysema (CPFE) commonly coexists with connective tissue diseases (CTD), such as rheumatoid arthritis (RA). However, the risk factors contributing to the development of CTD-CPFE remain largely unidentified. This study aimed to characterize CPFE using a large cohort of consecutive RA patients and to elucidate potential risk factors associated with RA- CPFE development.

**Methods:**

A total of 976 RA patients were enrolled in this cross-sectional study to characterize RA-CPFE. Multiple logistic analyses were conducted to identify potential risk factors for RA-CPFE development. Patient IgG and IgM autoantibodies to primary human bronchial epithelial cells (HBEC) from healthy donors were assessed using flow cytometry.

**Findings:**

Among the 976 RA patients, 414 (42.4%) developed interstitial lung disease (ILD), with 74 (7.6%) experiencing CPFE. In comparison to RA-CPFE patients with centrilobular or paraseptal emphysema, those with panacinar emphysema had higher emphysema scores and decreased pulmonary function parameters. Multiple logistic regression analysis revealed that male gender, cigarette smoking, occupational exposure to dust, high ILD score, high rheumatoid factor (RF) titers, and the presence of anti-SSA were associated with an increased risk for RA-CPFE. Additionally, levels of IgG and IgM autoantibodies to HBEC were elevated in RA-CPFE patients compared to healthy controls and positively correlated with RF levels.

**Interpretation:**

This study is the first to demonstrate the association of RA-CPFE with high titer of RF and the presence of autoantibodies against HBEC, suggesting a link between autoimmunity to the lung and RA-CPFE.

## Introduction

Emphysema and pulmonary fibrosis are distinct entities with differing radiological, pathological, and prognostic characteristics. The coexistence of emphysema and idiopathic pulmonary fibrosis (IPF) was initially reported in 1948 (1). Recognizing the increasing prevalence of this coexistence, Cottin et al. defined it as a distinct entry, namely combined pulmonary fibrosis and emphysema (CPFE) in 2005 (2). Recently, an official statement from ATS/ERS/JRS/ALAT proposed considering CPFE as a syndrome due to its distinct clinical features and pathogenesis (3). The prevalence of CPFE in patients with IPF varies significantly depending on the population and definition used (4–6). Notably, the majority of CPFE patients are males and smokers, as evidenced by a cohort where 60 out of 61 patients are male and all are current or former smokers (2), suggesting a crucial role of male sex and cigarette smoking in CPFE development.

Apart from IPF, emphysema is also reported to coexist with connective tissue disease-associated interstitial lung diseases (CTD-ILD), termed as CTD-CPFE (7). CTD encompasses a group of rheumatoid autoimmune disorders like rheumatoid arthritis (RA) and systemic sclerosis (SSc). The presence of CPFE increases mortality risk both in RA (8) and SSc (9) patients. Notably, compared to IPF-associated CPFE, CTD-CPFE patients are younger, more often females and never smokers, with higher inflammation and germinal center scores, but fewer fibrotic foci, lower emphysema scores, and better survival (10), indicating different pathogenic mechanisms between the two CPFE types. Despite its significant implications, CTD-CPFE has not been extensively studied, possibly due to the lower prevalence of rheumatoid disorders.

In this study, we enrolled 976 consecutive patients with RA with two main objectives. First, we aimed to characterize RA-CPFE in terms of prevalence and subtypes. Second, we aimed to identify potential risk factors associated with the development of RA-CPFE.

## Patients and methods

### Studied subjects

In this cross-sectional study, all patients with rheumatoid arthritis (RA) were recruited from the Department of Rheumatology at the First Affiliated Hospital of Xiamen University, Xiamen, China. The diagnosis of RA followed the 2010 RA classification criteria proposed by the American College of Rheumatology/European League Against Rheumatism (11). To avoid potential bias, consecutive patients were recruited during time period of between 2017 and 2022. Patients were excluded if they developed other connective tissue diseases (CTD) or had other pulmonary diseases that might lead to the development of lung fibrosis, such as pulmonary infection, pneumoconiosis, bronchiectasis and tuberculosis. High-resolution computed tomography (HRCT) was performed on all patients for the diagnosis of interstitial lung disease (ILD), emphysema and CPFE. ILD was defined by the presence of interstitial fibrosis in the lungs, manifested as reticulation cysts, thickening of the interlobular septum, ground-glass opacity, honeycomb cysts, or traction bronchiectasis (12). Diagnosis of CPFE followed the research definition proposed by the 2022 official ATS/ERS/JRS/ALAT research Statement (3). This study was conducted in accordance with the 1964 Declaration of Helsinki and its subsequent amendments or similar ethical standards. This study has obtained approval from the Ethics Committee of the First Affiliated Hospital of Xiamen University, Xiamen, China (No. 2021064 and No. 2023031). All participants in this study provided written informed consent.

### Data collection

Demographic and clinical characteristics including age, sex, body mass index (BMI), education status, residential location, smoking history, occupational exposure to dust, duration of RA and clinical presentations were retrieved from medical records. Some RA patients underwent pulmonary function tests (PFT). Specific PFT parameters included percentage predicted forced vital capacity (FVC%), percentage predicted forced expiratory volume in 1 second (FEV1%), FEV1/FVC ratio, residual volume (RV)/total lung capacity (TLC) ratio, and percentage predicted diffusing capacity of the lung for carbon monoxide (DLCO%). Laboratory parameters such as levels of circulating IgA, IgG, IgM, complement component 3 (C3), C4, and autoantibodies including antinuclear antibody (ANA), rheumatoid factor (RF), anti-cyclic citrullinated peptide (CCP), anti-SSA, anti-SSB, and anti-Ro52, determined at the patients’ admission for diagnosis, were also retrieved.

### HRCT evaluation

HRCT of the chest were reviewed and analyzed independently by two thorax radiologists who were blinded to clinical data to assess the emphysema score, ILD score and CPFE score. Evaluation of emphysema score was conducted by examining 3 anatomical levels of both left and right lung, namely upper, middle and lower lung fields. Emphysematous changes in each field were scored based on the criteria proposed by Goddard et al. (13). ILD score, reflecting the severity of pulmonary fibrosis, was evaluated in the same six lung fields based on the percentage of affected area (14).

### Determination of autoantibodies directed against human bronchial epithelial cells

Primary human bronchial epithelial cells (HBEC) were obtained from Cellverse Bioscience Technology Co., Ltd., Shanghai, China. Binding of serum IgG and IgM autoantibodies to HBECs was assessed using flow cytometry. Confluent HBECs were detached using Accutase™ Cell Detachment Solution (Biolegend, San Diego, CA) which helps to gently detach cells while largely maintaining surface antigen integrity (15), washed, and suspended in PBS with Fc block and live dead dye. Serum samples (diluted 1:50 with PBS with 0.1% BSA) were added and incubated for 20 minutes at 4°C, followed by washing and incubation with APC anti-human IgG Fc and Alexa Fluor^®^ 488 anti-human IgM or their isotype controls (Biolegend, San Diego, CA). After additional washing, cells were fixed and analyzed using a flow cytometer (BD, USA). Mean fluorescence intensity (MFI) values of the Alexa Fluor^®^ 488 anti-human IgM and APC anti-human IgG Fc were regarded as levels of IgM and IgG autoantibodies, respectively.

### Statistical analyses

All statistical analyses were performed using GraphPad Prism Software (version 10.0.2). The Kolmogorov-Smirnov test was utilized to assess the normal distribution of variables. For normally distributed quantitative data, the student t-test was employed to determine statistical significance; otherwise, the Mann–Whitney *U* test was applied. One-way analysis of variance (ANOVA) was utilized for comparisons involving more than two group means. Spearman’s rank correlation was applied to determine relationship between two quantitative variables. For qualitative variables, statistical significance was determined using the chi-squared test or Fisher’s exact test. The Chi-square test for trend was used to evaluate the trend of increasing antibody prevalence. A *p*-value less than 0.05 was considered statistically significant.

## Results

### Demographic characterization of patients and prevalence of RA-CPFE

**Figure 1:**
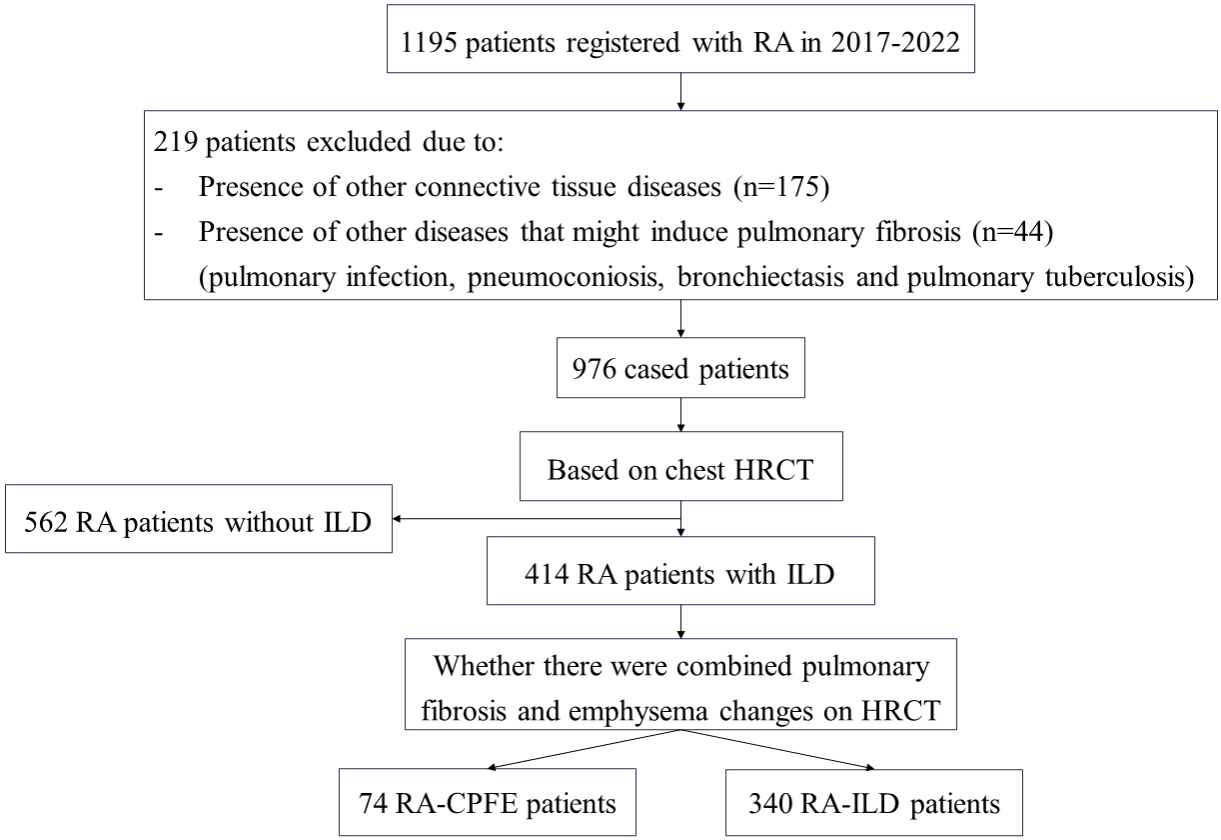
Flow diagram describing recruitment and progress of participants to this study.

In total, 1195 consecutive patients with RA were reviewed in the present study. After excluding patients developed other CTD or had comorbidity of pulmonary diseases that may cause lung fibrosis, 976 patients with RA (278 males and 698 females) were recruited in this study (**Figure 1**). Among the 976 patients, 414 (42.4%) exhibited ILD, and 74 (7.6%) developed CPFE (**Table 1**). The mean age of patients with RA, RA-ILD and RA-CPFE were 57.8, 61.9, and 64.2 years, respectively. Compared to entire RA patients and patient with RA-ILD, patients with RA-CPFE showed a decreased body mass index (BMI). When patients were categorized into males and females, the prevalence of CPFE was significantly higher in males than in females in both the entire RA patients (21.6% vs 2.0%, *p*<0.0001) and the subgroup of RA-ILD patients (39.2% vs 5.4%, p<0.0001). When patients were categorized according to their smoking status, the prevalence of CPFE was significantly higher in smokers than never -smokers in both the entire RA patients (27.5% vs 2.5%, *p*<0.0001) and in patients with RA-ILD (45.7% vs 6.5%, *p*<0.0001).

**Table 1:** Demographic characterization of patients with RA, RA-ILD and RA-CPFE.

	Total RA	RA-ILD	RA-CPFE
Number (%)	976 (100)	414 (42.4)	74 (7.6)
Age (mean ± SD	57.8 ± 11.5	61.9 ± 9.7	64.2 ± 7.8
BMI	22.1 ± 3.28	22.4 ± 3.38	20.9 ± 2.88
Male/Female	278/698	153/261	60/14
Smoker/never smoker	156/820	94/320	43/21

### Characterization of patients with RA-CPFE

**Figure 2:**
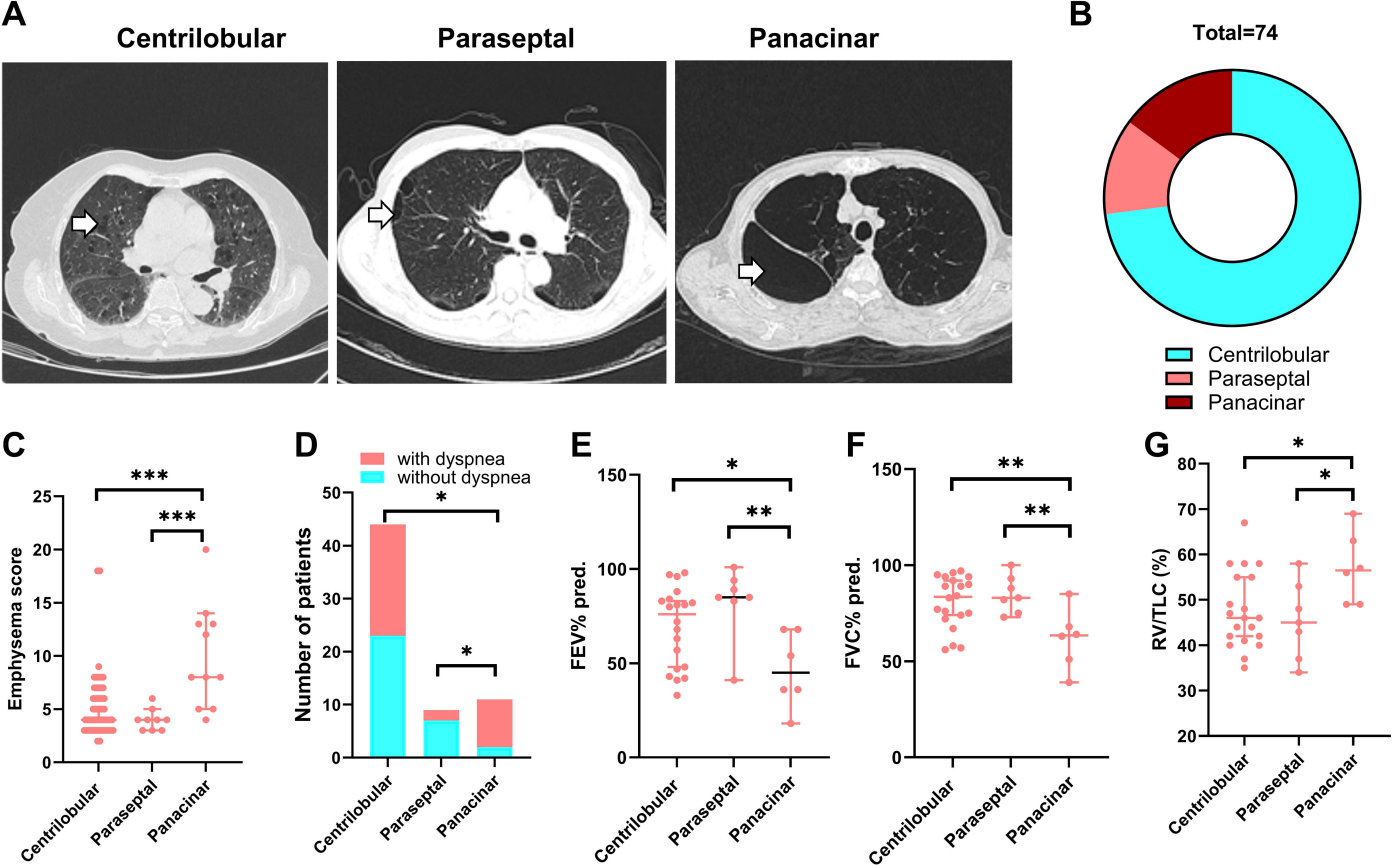
Comparison of three subgroups of patients RA-CPFE. RA-CPFE patients were categorized into three subgroups according types of emphysema foci, centrilobular, parasepital and panacinar. **(A)** Representative HRCT chest images of the three types of emphysema foci. White arrows indicate the emphysema foci. **(B)** The pie chart of the composition of the three types of emphysema foci in 74 patients with CPFE. Comparison of the three subgroups of RA-CPFE patients on emphysema score **(C)**, proportion of patients with dyspnea **(D)**, FEV1% predicted **(E)**, FVC% predicted **(F)** and RV/TLC **(G)**. *p<0.05, **<0.01, ***p<0.001.

We then characterized the 74 patients with RA-CPFE. Among them, 60 (81.1%) exhibited reticulation cysts, 28 (37.8%) showed thickening of the interlobular septum, 26 (35.1%) presented with ground-glass opacity, 14 (18.9%) had honeycomb cysts, and 11 (14.9%) showed traction bronchiectasis (**Supplementary Table 1**). Based on the type of emphysema foci, these patients were categorized into three groups. The first group comprised 54 patients with centrilobular emphysema, the second group included 11 patients with panacinar emphysema, and the third group consisted of 9 patients with paraseptal emphysema (**Supplementary Table 2**, **Figures 2A, B**). Patients with panacinar emphysema showed a higher median emphysema score compared to patients with centrilobular (8.0 vs 4.0, *p*=0.0002) or paraseptal (8.0 vs 4.0, *p*=0.0004) emphysema (**Figure 2C**). Accordingly, the frequency of dyspnea in patients with panacinar emphysema was 81.8%, which was significantly higher than in the centrilobular (40.7%, *p*=0.019) and paraseptal (22.2%, *p*=0.022) groups (**Figure 2D**). Among the 74 patients with RA-CPFE, 33 underwent lung function tests, including 20, 7, and 6 patients from the centrilobular, paraseptal, and panacinar groups, respectively. Compared to RA-CPFE patients with panacinar type, patients with centrilobular and paraseptal types showed significantly higher levels of predicted FEV1% and FVC% and RV/TLC value (**Supplementary Table 3**, **Figures 2E–G**).

Given that some patients with RA-CPFE were females or never smokers, we conducted a stratified analysis based on sex and smoking history. Compared to male patients with RA-CPFE, the corresponding group of female patients consisted of significantly less smokers (7.1% *vs* 70%, *p*<0.0001). Moreover, female patients with RA-CPFE exhibited a higher FEV1/FVC (%) than male patients (**Supplementary Table 3**). When RA-CPFE patients were categorized into two groups based on smoking history, smokers exhibited a higher emphysema score than female patients (**Supplementary Table 4**).

To further characterize RA-CPFE, we determined which demographic, clinical, and laboratory features were associated with the severity of RA-CPFE in terms of ILD, emphysema, and CPFE scores (**Table 2**, **Supplementary Figure 1**). The strongest correlation was observed between CPFE score and percentile predicted DLCO (r=-0.84, *p*=1.66x10^-09^). In addition to DLCO, other pulmonary function parameters including FEV1%, FEV1/FVC, and RV/TLC were significantly correlated with CPFE score. Similarly, emphysema score was significantly correlated with FEV1%, FEV1/FVC, RV/TLC, and DLCO. Additionally, an inverse correlation between emphysema score and BMI in patients with RA-CPFE was observed. In contrast, ILD score was only significantly associated with DLCO.

**Table 2:** Correlation of ILD score and emphysema score with demographic, clinical and laboratory characteristics in patients with RA-CPFE.

	ILD score		Emphysema score		CPFE score	
	r	*P* values	r	*P* values	r	*P* values
Age	0.10	n.s.	0.01	n.s.	0.13	n.s.
BMI	0.07	n.s.	-0.31	0.0064	-0.21	n.s.
RA duration	-0.08	n.s.	-0.16	n.s.	-0.14	n.s.
RF	-0.04	n.s.	0.06	n.s.	-0.02	n.s.
anti-CCP	-0.05	n.s.	0.10	n.s.	-0.03	n.s.
C3	-0.12	n.s.	0.06	n.s.	-0.09	n.s.
C4	-0.11	n.s.	0.23	n.s.	0.00	n.s.
IgA	0.17	n.s.	0.13	n.s.	0.26	n.s.
IgG	0.10	n.s.	0.01	n.s.	0.09	n.s.
IgM	0.03	n.s.	-0.06	n.s.	-0.02	n.s.
FEV1% predicted*	-0.25	n.s.	-0.50	0.003	-0.48	0.004
FVC% predicted*	-0.27	n.s.	-0.06	n.s.	-0.23	n.s.
FEV1/FVC (%)*	-0.16	n.s.	-0.49	0.004	-0.40	0.023
RV/TLC (%)*	0.21	n.s.	0.43	0.012	0.38	0.029
DLCO% predicted*	-0.67	2.88x10^-05^	-0.51	0.003	-0.84	1.66x10^-09^

*Pulmonary functional test parameters include 33 patients with RA-CPFE. n.s., not significant.

### Clinical and laboratory factors associated with RA-CPFE

**Figure 3:**
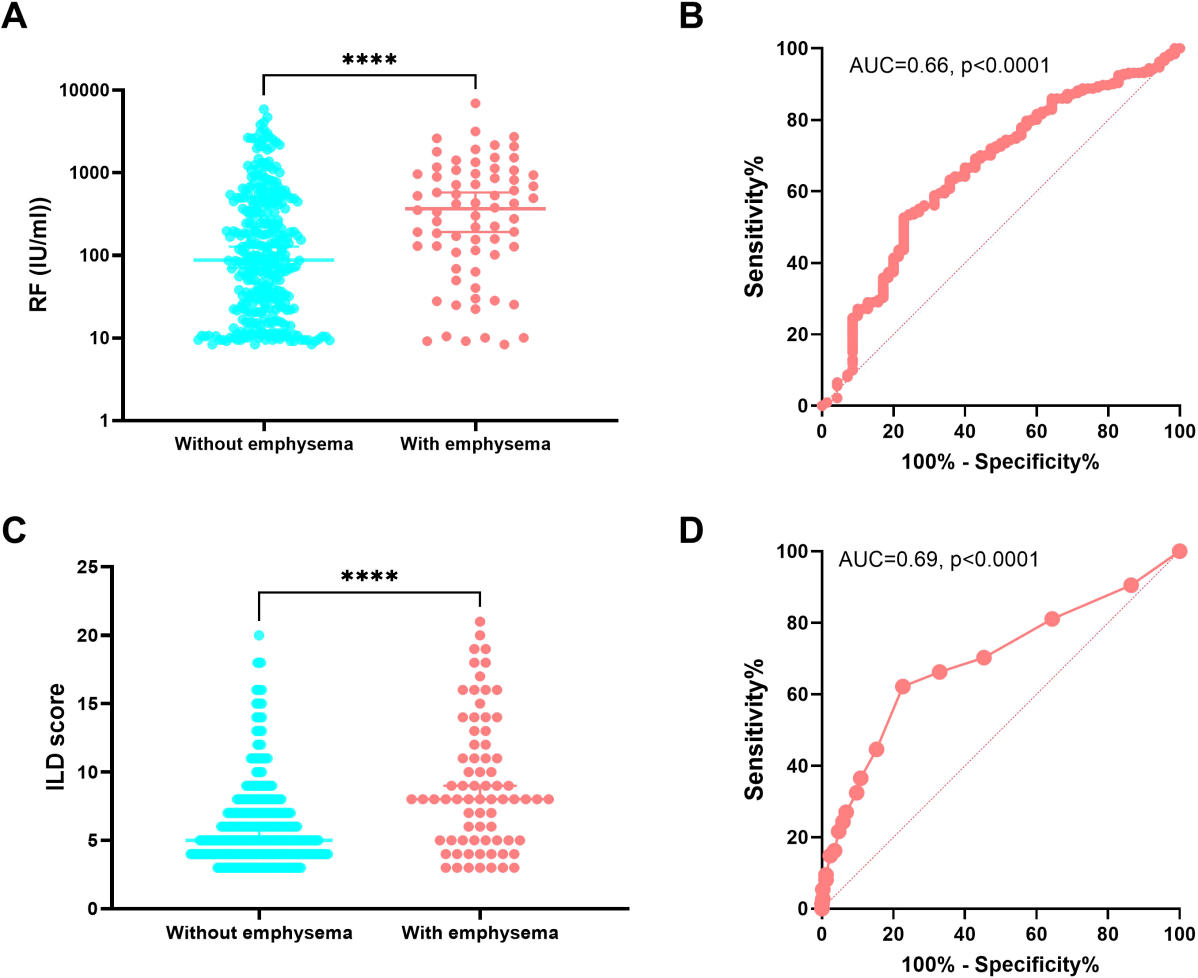
Association of the presence of emphysema in RA-ILD with rheumatoid factor and ILD score. Comparison of levels of RF **(A)** and ILD score **(C)** between RA-ILD patients with and without emphysema. Receiver operating characteristic (ROC) curve analysis of levels of RF **(B)** and ILD score **(D)** and their areas under curve (AUC) of RA-ILD patients with emphysema compared to RA-ILD patients without emphysema. ****p<0.0001.

To identify factors that might be associated with RA-CPFE, we compared RA-ILD patients with and without emphysema. RA-ILD patients with emphysema were older, had a lower BMI, more often males and smokers and had higher rates of occupational exposure to dust. In terms of clinical features, RA-ILD patients with emphysema exhibited a higher median ILD score (8 *vs* 5, *p*<0.0001) and had a higher prevalence of comorbidities such as gastroesophageal reflux and lung cancer. Additionally, impaired lung function was observed in RA-ILD patients with emphysema compared to those without emphysema, as indicated by lower levels of FEV1% and FEV1/FVC, higher levels of RV/TLC, and lower levels of percent predicted DLCO%. RA-ILD patients with and without emphysema differed in several laboratory characteristics. Notably, RA-ILD patients with emphysema exhibited higher median levels of rheumatoid factors (RF) (367 IU/ml *vs* 87 IU/ml, *p*<0.0001) and anti-CCP antibodies (157.6 RU/ml *vs* 73.9 RU/ml, *p*=0.0013) than those without emphysema. Increased frequencies of two other autoantibodies, ANA and SSA, were found in RA-ILD patients with emphysema compared to those without emphysema. Moreover, RA-ILD patients with emphysema showed lower levels of circulating complement C3 compared to those without emphysema, but there was no significant difference in levels of C4 (**Table 3**, **Figure 3**).

**Table 3:** Comparison of demographic, clinical and laboratory characteristics between RA-CPFE and RA-ILD.

	RA-ILD with emphysema (n=74)	RA-ILD without emphysema (n=340)	*P* values
Sex (male/female)	60/14	93/247	<0.0001
Age (in years)	64.23 ± 7.84	61.37 ± 9.94	0.021
BMI (kg/m^2^)	20.85 ± 2.88	22.78 ± 3.39	<0.0001
Education (≥9 years/<9 years)	27/47	92/248	n.s.
Residential location (Urban/Rural)	28/46	153/187	n.s.
Duration of RA (in years)	7.5 (2.0-15.0)	3.0 (1.0-10.0)	0.0031
Smoker (n/%)	43 (58.1)	51 (15.0)	<0.0001
Pack years	40 (30-50)	40 (30-50)	n.s.
Occupational exposure to toxic particles and gas, n (%)	16 (21.6)	27 (7.9)	0.0004
Clinical presentations			
Joint tenderness, n (%)	70 (94.6)	332 (97.6)	n.s.
Morning stiffness, n (%)	53 (71.6)	259 (76.2)	n.s.
Raynaud’s phenomenon, n (%)	15 (20.3)	66 (19.4)	n.s.
Chronic cough, n (%)	27 (36.5)	133 (39.1)	n.s.
Dyspnea, (n/%)	33 (44.6)	120 (35.3)	n.s.
Treatment			
Steroids	63 (87.6%)	298 (85.1%)	n.s.
Immosuppressor	74 (100%)	337 (99.1%)	n.s.
Home oxygen therapy	18 (24.3%)	45 (13.2%)	0.0161
Comorbidities			
Allergic diseases, n (%)	2 (2.7)	19 (5.6)	n.s.
Gastroesophageal reflux, n (%)	16 (21.6)	30 (8.8)	0.0014
Hypertension, n (%)	18 (24.3)	100 (29.4)	n.s.
Diabetes, n (%)	6 (8.1)	43 (12.6)	n.s.
Coronary heart disease, n (%)	6 (8.1)	22 (6.5)	n.s.
Lung cancer, n (%)	4 (5.4)	3 (0.9)	0.0215
ILD score	8 (5, 12)	5 (4, 7)	<0.001
Laboratory characteristics			
ANA, n (%)	30 (40.5)	91 (27.9)	0.0378
anti-SSA, n (%)	11 (14.9)	19 (5.8)	0.0078
anti-SSB, n (%)	2 (2.7)	3 (0.9)	n.s.
anti-Ro52, n (%)	9 (12.2)	36 (11.1)	n.s.
RF (IU/mL)	367 (113, 945)	87 (22, 417)	<0.0001
anti-CCP(RU/mL)	157.6 (43.4, 200.0)	73.9 (19.4, 200.0)	0.0013
C3 (g/L)	1.05 (0.90, 1.27)	1.17 (1.05, 1.36)	0.0012
C4 (g/L)	0.24 (0.18, 0.31)	0.25 (0.20, 0.31)	n.s.
IgA (g/L)	3.37 (2.52, 4.35)	2.92 (2.28, 3.86)	n.s.
IgG (g/L)	12. (10.5, 15.6)	13.2 (11.0, 16.3)	n.s.
IgM (g/L)	1.45 (0.96, 1.89)	1.29 (0.91, 1.80)	n.s.
Lung function test parameters^*^			
FEV1% predicted	67.9 ± 22.9	81.5 ± 17.8	0.0036
FCV1% predicted	78.2 ± 15.2	76.7 ± 14.9	n.s.
FEV1/FVC (%)	65.0 ± 17.4	82.9 ± 10.6	<0.0001
RV/TLC (%)	48.9 ± 9.0	42.6 ± 10.6	0.0066
DLCO% predicted	45.7 ± 18.9	59.5 ± 21.8	0.0040

*Pulmonary functional test parameters include 33 patients with RA-CPFE and 48 patients with RA-ILD. Reference range: RF:0-15.9 IU/mL, anti-CCP:0-5 RU/mL. C3:0.9-1.8 g/L, C4:0.1-0.4 g/L, IgA:0.7-4 g/L, IgG:7-16 g/L, IgM:0.4-2.3 g/L.

To better understand the association of variables with RA-CPFE, six characteristics that showed a highly significant association with the presence of emphysema in patients with RA-ILD were selected for further evaluation. These included sex, smoking status, occupational exposure to toxic particles and gas, RF, SSA, and ILD score. To facilitate multiple logistic regression analysis, two quantitative variables, RF and ILD, were converted into qualitative variables: RF^high^ (>100 IU/ml) *vs* RF^low^ (≤100 IU/ml) and ILD^high^ (>7) *vs* ILD^low^ (≤7), using the cut-off value defined as the point which showed maximal Youden index (Sensitivity - (1- Specificity)) in ROC curve analysis (**Figure 3**). The odds ratios (OR) calculated from the multiple logistic regression analysis for these six variables are summarized in **Table 4**. In addition to confirming previously established risk factors for RA-CPFE, such as male sex, cigarette smoking, and occupational exposure to toxic particles and gases, multiple logistic regression analysis identified significant associations between RA-CPFE and elevated levels of ILD (OR = 5.80, 95% CI = 3.05–11.41, p < 0.0001), high RF levels (OR = 2.08, 95% CI = 1.04–4.33, p = 0.043), and the presence of anti-SSA antibodies (OR = 3.10, 95% CI = 1.10–8.51, p = 0.03).

**Table 4:** Multiple logistic regression analysis for factors associated with development of emphysema in patient with RA-ILD.

	|Z|	VIF	OR (95% CI)	*P* values
Sex	9.35	2.202	4.66 (1.96-11.21)	0.0005
Cigarette smoke	3.48	2.039	2.72 (1.23-6.19)	0.015
Occupational exposure to toxic particles and gas	2.44	1.055	2.40 (1.01-5.62)	0.045
High titer of RF (>100IU/ml)	2.01	1.125	2.08 (1.04-4.33)	0.043
anti-SSA	2.02	1.019	3.10 (1.10-8.51)	0.030
High ILD score (>7)	2.17	1.058	5.80 (3.05-11.41)	<0.0001

### Autoantibodies against primary human bronchial epithelial cells in patients with RA-CPFE

Considering that both RF- and anti-SSA-levels were elevated in RA-ILD-patients with emphysema as compared to patients with solely RA-ILD, we hypothesized that autoimmunity to the lung might contribute to the development of RA-CPFE. Given that autoantibodies to extracellular antigens, different from intracellular ones, are potentially pathogenic (16, 17), we determined circulating autoantibodies to extracellular membrane antigens of primary HBEC. Serum samples from 35 healthy subjects, 25 RA-ILD patients without emphysema, and 12 patients with RA-CPFE were collected for investigation (**Supplementary Table 5**).

**Figure 4:**
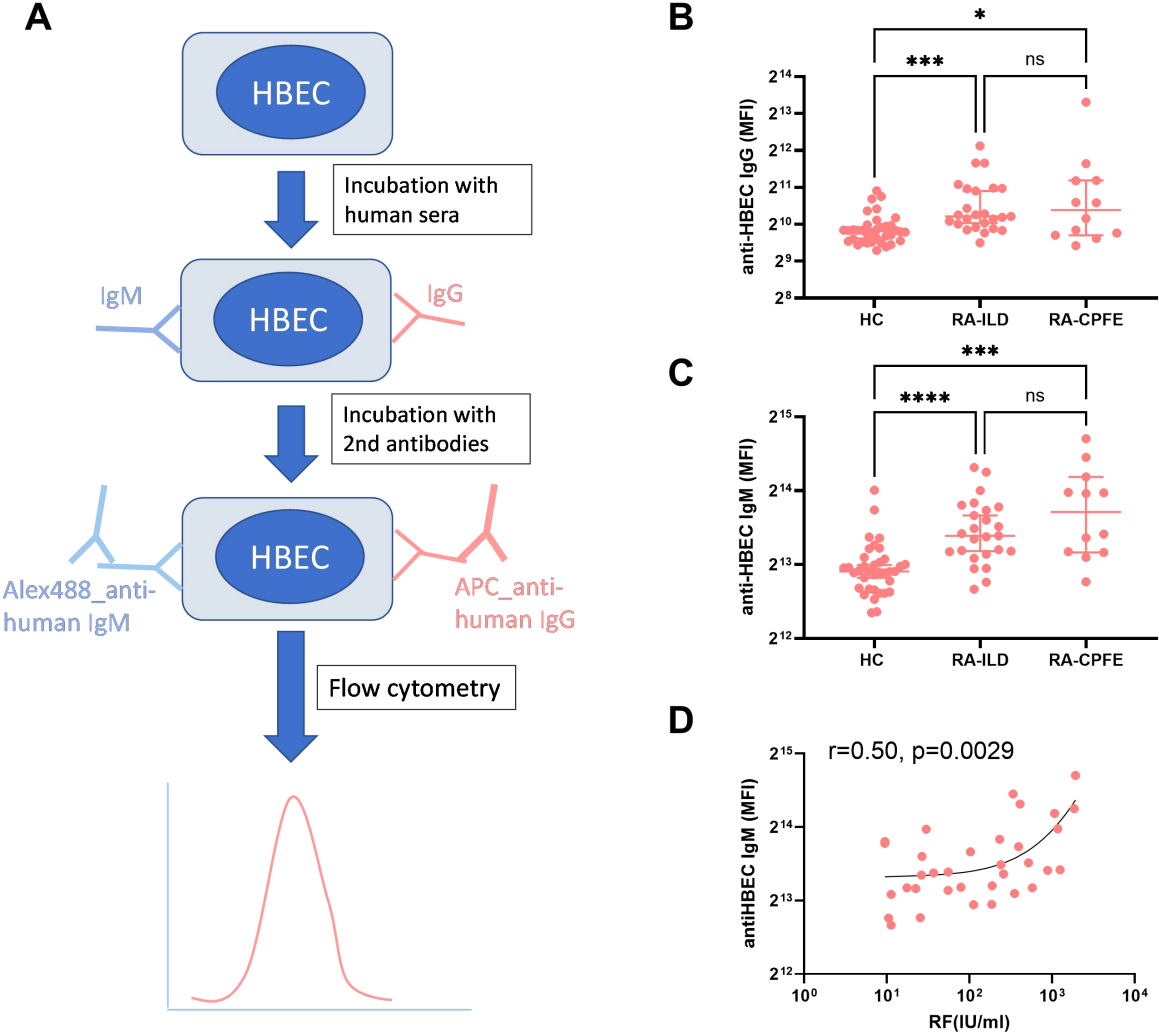
Association of RA-CPFE with autoantibodies to primary HBEC. **(A)** A schematic flowchart of detection of IgG and IgM autoantibodies to extracellular antigens of primary human bronchial epithelial cell (HBEC). Comparison of levels of IgG **(B)** and IgM **(C)** autoantibodies to primary HBEC between healthy control (HC), RA-ILD patients without emphysema and RA-ILD patients with emphysema. **(D)** Correlation between IgM autoantibody against HBEC and rheumatoid factor (RF) in patients with RA-ILD. *p<0.05, ***p<0.001, ****p<0.0001.

Levels of IgG and IgM autoantibodies to primary HBEC were determined using flow cytometry and represented as mean fluorescence intensity (MFI) (**Figure 4A**). Both RA-ILD patients without emphysema and patients with RA-CPFE showed significantly higher levels of IgG autoantibodies to HBEC than healthy subjects (**Figure 4B**). Similarly, levels of IgM autoantibodies to HBEC in patients with RA-ILD without emphysema and patients with RA-CPFE were significantly increased compared to healthy subjects (**Figure 4C**). Finally, we determined whether IgG and IgM autoantibodies are correlated with clinical and laboratory characteristics in RA-ILD patients with and without emphysema. Notably, BMI was inversely correlated with levels of anti-HBEC IgG (r=-0.44, *p*=0.0067) and levels of anti-HBEC IgM (r=-0.40, *p*=0.0132) (**Supplementary Table 6**). Moreover, a positive correlation was observed between levels of RF and IgM autoantibodies to HBEC (r=0.50, *p*=0.0029) (**Figure 4D**).

## Discussion

In this study, we investigated RA-CPFE in 976 consecutive patients with RA, constituting the largest cohort studied for RA-CPFE research to date. This provided an unprecedented opportunity to estimate the prevalence of CPFE in patients with RA. Among the 976 patients, 74 (7.6%) developed CPFE. This prevalence is comparable to the 8% value observed in 150 consecutive RA patients in a Caucasian population (18), indicating consistent prevalence across different populations.

It has been well established that the presence of pulmonary fibrosis and emphysema impairs lung function (19–22). In addition, emphysema and fibrosis scores are correlated with functional parameters of obstruction and restriction, respectively in patients with CPFE (23). However, the correlation of the extent of fibrosis and emphysema with pulmonary function in patients with CTD-CPFE has not been investigated. In the current study, we determined the correlation of the extent of fibrosis and emphysema with pulmonary parameters in patients with RA-CPFE. Interestingly, we found that the ILD score was inversely correlated only with DLCO in patients with RA-CPFE, suggesting that lung fibrosis affects exercise capacity. In contrast, the emphysema score was not only inversely correlated with DLCO but also significantly correlated with FEV1%, FEV1/FVC, and RV/TLC in patients with RA-CPFE. This suggests that emphysema impairs both exercise capacity and air diffusion capacity. Furthermore, our observations indicate that RA-CPFE patients of panacinar type exhibit a higher emphysema score, lower levels of predicted FEV1% and FVC%, and a higher ratio of RV/TLC compared to those of centrilobular and paraseptal types.

The multiple logistic regression analysis demonstrated that a high ILD score is a strong and independent factor associated with an increased risk of developing CPFE in patients with RA. This finding aligns with observations in patients with SSc, where SSc-CPFE patients show a higher score of semiquantitative assessment of ILD extension detectable in chest CT than SSc-ILD patients without emphysema (9). However, it’s important to note that the association of the extent of ILD with CPFE is not always consistent in previous studies. For instance, Jacob et al. demonstrated that ILD extent is comparable between RA-ILD patients with and without emphysema (8). Additionally, Antoniou and colleagues reported that although IPF patients with emphysema show a higher score of pulmonary fibrosis than IPF patients without emphysema, no significant difference has been observed in the fibrosis score between RA-ILD patients with and without emphysema (24). This discrepancy warrants further investigations in future studies to elucidate the underlying factors contributing to these differences.

The current study reveals a noteworthy association between the development of RA-CPFE and two autoantibodies, RF and anti-SSA. This finding is significant, particularly in light of previous studies suggesting that never smokers can develop CPFE in patients with CTD, indicating that CTD itself may be a risk factor for CPFE development (8, 9, 25). For the first time, our study delves into the relationship between autoantibodies and RA-CPFE, demonstrating that a high titer of RF and the presence of anti-SSA are linked to an increased risk of RA-CPFE. This discovery aligns with earlier observations indicating an elevated incidence of autoimmune markers in patients with CPFE compared to those with IPF (4). Considering that autoantibodies such as RF and anti-CCP often precede the clinical diagnosis of RA (26, 27), it is plausible to speculate that autoimmunity may contribute to the pathogenesis of emphysema in patients with RA-ILD, ultimately leading to the development of RA-CPFE.

The presence of autoantibodies to primary HBEC in patients with RA-CPFE further supports the association of autoimmunity with RA-CPFE, providing evidence for the link between RA-CPFE and autoimmunity to the lung. This association between autoimmunity and emphysema has been extensively explored and firmly established in chronic obstructive pulmonary disease (COPD) (28, 29). It has been shown that COPD patients exhibit elevated levels of autoantibodies to extracellular antigens compared to healthy individuals (30). Additionally, autoantibodies targeting pulmonary epithelial cells have been identified in COPD patients, with the potential to induce cytotoxic effects (31), suggesting a possible role in emphysema formation. Consequently, the potential pathogenicity of autoantibodies to HBEC in RA-CPFE warrants further investigation. In addition to humoral autoimmunity, autoreactive T cells, particularly those targeting elastin, an extracellular protein crucial for lung integrity, have been implicated in contributing to the development of COPD and its animal model (29, 32). Therefore, exploring the involvement of autoreactive T cells in the lung in the context of RA-CPFE holds significance and merits further investigation.

The findings of the present study have significant clinical implications, particularly for low- and middle-income countries. In many low-income settings, access to advanced diagnostic tools such as CT scans is limited and typically available only to a small proportion of patients (33). Consequently, the observed associations of RA-CPFE with high titers of RF, anti-SSA antibodies, male gender, cigarette smoking, and occupational dust exposure could inform the prioritization of patients for HRCT imaging. This targeted approach may enhance the efficiency and appropriateness of HRCT utilization in these resource-constrained environments. Furthermore, smoking cessation can be recommended as an effective, low-cost intervention for patients with RA-CPFE, potentially mitigating disease progression and associated complications.

This study is subject to a major limitation. As it is cross-sectional in nature, both exposure and outcome are assessed concurrently at a specific time point, precluding the evaluation of changes over time. Consequently, establishing a clear temporal association between exposure and outcome is challenging. However, considering that autoantibodies, including RF, precede the diagnosis of RA (26, 27), their association with RA-CPFE can still be considered indicative of a potential role of autoimmunity in the pathogenesis of the disease.

In conclusion, this study highlights a positive association of male gender, cigarette smoking, occupational exposure to dust, and the extent of fibrosis with emphysema in patients with RA-ILD, supporting their roles as risk factors for the development of CPFE. Furthermore, for the first time, this study demonstrates an association of RA-CPFE with autoantibodies such as RF, anti-SSA, and anti-HBEC, linking autoimmunity to the lung with the disease.

## Data Availability

The original contributions presented in the study are included in the article/**Supplementary Material**. Further inquiries can be directed to the corresponding authors.
