# Supplementary material for: Association of combined pulmonary fibrosis and emphysema in rheumatoid arthritis with high titer of rheumatoid factor and autoimmunity to the lung
Source: Front Immunol. 2025 Feb 5;16:1514552. doi: 10.3389/fimmu.2025.1514552 (PMC11836040; doi:10.3389/fimmu.2025.1514552)
Supplement: Supplementary file 1 [file DataSheet1.docx]

**Supplementary information**

**Supplementary table 1. ILD features and pattern of emphysematous foci in patients with RA-CPFE**

|  | **Number of patients** | **% of total** |
| --- | --- | --- |
| **ILD features** |  |  |
| Thickening of the interlobular septum | 28 | 37.8% |
| Ground-glass opacity | 26 | 35.1% |
| Reticulation cysts | 60 | 81.1% |
| Honeycomb cysts | 14 | 18.9% |
| Traction bronchiectasis | 11 | 14.9% |
| **Pattern of Emphysematous foci** |  |  |
| Centrilobular | 54 | 73.0% |
| Paraseptal | 9 | 12.2% |
| Panacinar | 11 | 14.9% |

**Supplementary table 2. Three types of emphysema foci in patients with RA-CPFE**

|  | **Centrilobular** | **Panacinar** | **Paraseptal** |
| --- | --- | --- | --- |
| Number | 54 | 11 | 9 |
| Sex (female/male) | 10/44 | 2/9 | 1/8 |
| Age (in years) | 64.0 ± 8.7 | 65.6 ± 6.4 | 63.9 ± 3.3 |
| BMI (kg/m^2^) | 21.1 ± 2.7 | 19.7 ± 3.1 | 20.9 ± 3.5 |
| Education (≥9 years/＜9 years) | 21/33 | 5/6 | 1/8 |
| Residential location (Urban/ Rural) | 22/33 | 5/6 | 1/8 |
| Duration of RA (in years) | 4.5 (1.75-10.0) | 1.0 (1.0-11.0) | 3.0 (2.5-5.5) |
| Smoker (n/%) | 30 (55.5%) | 8 (72.7%) | 5 (55.5%) |
| Pack years | 4.5 (1.75, 10.0) | 1.0 (1.0, 11.0) | 3.0 (2.5, 5.5) |
| Occupational exposure to dust, n (%) | 11 (20.4%) | 1 (9.1%) | 4 (44.4%) |
| Clinical presentations |  |  |  |
| Joint tenderness, n (%) | 51 (94.4%) | 10 (91.0%) | 9 (100%) |
| Morning stiffness, n (%) | 37 (68.5%) | 8 (72.7%) | 8 (88.9%) |
| Raynaud's phenomenon, n (%) | 10 (18.5%) | 2 (18.8%) | 3 (33.3%) |
| Chronic cough, n (%) | 17 (31.4%) | 7 (63.6%) | 3 (33.3%) |
| Dyspnea, (n/%) | 22 (40.7%) * | 9 (81.8%) | 2 (22.2%) * |
| Treatment |  |  |  |
| Steroids | 48 (88.9%) | 10 (91%) | 5 (55.6%) |
| Immosuppressor | 54 (100%) | 11 (100%) | 9 (100%) |
| Home oxygen therapy | 9 (16.7%)** | 7 (63.6%) | 4 (44.4%) |
| Comorbidities |  |  |  |
| Allergic diseases, n (%) | 2 (3.7%) | 0 (0%) | 0 (0%) |
| Gastroesophageal reflux, n (%) | 11 (20.4%) | 2 (18.8%) | 3 (33.3%) |
| Hypertension, n (%) | 15 (27.8%) | 1 (9.1%) | 2 (22.2%) |
| Diabetes, n (%) | 5 (9.3%) | 0 (0%) | 1 (11.1%) |
| Coronary heart disease, n (%) | 1 (1.9%) | 1 (9.1%) | 0 (0%) |
| Lung cancer, n (%) | 3 (7.4%) | 0 (0%) | 1 (11.1%) |
| ILD score | 8.0 (5.0, 11.3) | 5.0 (4.0, 16.0) | 8.0 (7.5, 16.0) |
| Fibrosis feature |  |  |  |
| Reticulation cysts | 8 (88.9%) | 8 (72.7) | 44 (81.5%) |
| Thickening of the interlobular septum | 4 (44.4%) | 4 (36.4%) | 20 (37.0%) |
| Ground-glass opacity | 1 (11.1%) | 5 (45.5%) | 20 (37.0%) |
| Honeycomb cysts | 4 (44.4%) | 4 (36.4%) | 6 (11.1%) |
| Traction bronchiectasis | 1 (11.1%) | 4 (36.4%) | 6 (11.1%) |
| Emphysema score | 4.0 (3.0, 6.3) *** | 8.0 (5.0, 13.0) | 4.0 (3.0, 4.5) *** |
| CPFE score | 12 (10, 16.5) * | 18 (16, 24) | 13 (11, 20.5) |
| Lung function test parameters^§^ |  |  |  |
| FEV1% predicted | 69.2 ± 20.9* | 46.7 ± 20.1 | 82.6 ± 19.7** |
| FCV1% predicted | 80.8 ± 13.6** | 61.7 ± 15.6 | 84.9 ± 9.6** |
| FEV1/FVC (%) | 65.1 ± 13.9 | 54.7 ± 24.9 | 73.6 ± 16.9 |
| RV/TLC (%) | 47.6 ± 8.4* | 57.2 ± 7.9 | 45.4 ± 8.5* |
| DLCO% predicted | 50.4 ± 18.4 | 34.2 ± 14.3 | 48.3 ± 15.7 |
| Laboratory characteristics |  |  |  |
| ANA, n (%) | 22 (40.7%) | 5 (45.5%) | 3 (33.3%) |
| anti-SSA, n (%) | 8 (14.8%) | 3 (27.2%) | 0 (0%) |
| anti-SSB, n (%) | 2 (3.7%) | 0 (0%) | 0 (0%) |
| anti-Ro52, n (%) | 6 (11.1%) | 2 (18.8%) | 1 (11.1%) |
| RF (IU/mL) | 280 (58, 836) | 532 (130, 2090) | 716 (428, 1300) |
| anti-CCP(RU/mL) | 139.5 (42.2, 200) | 200 (82.8, 200) | 142.3 (25.5, 200) |
| C3 (g/L) | 1.11 ± 0.33 | 1.09 ± 0.15 | 0.98 ± 0.38 |
| C4 (g/L) | 0.23 (0.18, 0.31) | 0.29 (0.24, 0.34) | 0.18 (0.13, 0.27) |
| IgA (g/L) | 3.15 (2.44, 4.25) | 3.24 (2.37, 4.60) | 3.95 (3.15, 4.25) |
| IgG (g/L) | 12.1 (10.3, 16.0) | 13.6 (11.1, 15.9) | 13.3 (11.4, 14.8) |
| IgM (g/L) | 1.29 (0.93, 1.81) | 1.65 (1.24, 1.91) | 1.84 (1.40, 2.73) |

^§^Lung functional test parameters include 33 patients with RA-CPFE. **P*<0.05, ***P*<0.01, ****P*<0.001(compared to panacinar group).

**Supplementary table 3. Comparison of males and females in patients with RA-CPFE**

|  | **Male** | **Female** |
| --- | --- | --- |
| Number | 60 | 14 |
| Age (in years) | 64.6 ± 6.3 | 62.9 ± 12.7 |
| BMI (kg/m^2^) | 20.7 ± 2.7 | 21.1 ± 2.7 |
| Education (≥9 years/＜9 years) | 23/37 | 4/10 |
| Residential location (Urban/ Rural) | 22/38 | 6/10 |
| Duration of RA (in years) | 3.0 (1.0-9.0) | 5.5 (1.75-11.25) |
| Smoker (n/%) | 42 (70%) | 1 (7.1%) **** |
| Pack years | 40 (30, 50) | 30 (30, 30) |
| Occupational exposure to toxic particles and gas, n (%) | 13 (21.7%) | 3 (21.4%) |
| Clinical presentations |  |  |
| Joint tenderness, n (%) | 58 (96.6%) | 12 (85.7%) |
| Morning stiffness, n (%) | 45 (75%) | 8 (57.1%) |
| Raynaud's phenomenon, n (%) | 11 (18.3%) | 4 (28.6%) |
| Chronic cough, n (%) | 21 (35.0%) | 6 (42.9%) |
| Dyspnea, (n/%) | 26 (43.3%) | 7 (50%) * |
| Treatment |  |  |
| Steroids | 50 (83.3%) | 13 (92.9%) |
| Immosuppressor | 60 (100%) | 14 (100%) |
| Home oxygen therapy | 17 (28.3%) | 3 (28.3%) |
| Comorbidities |  |  |
| Allergic diseases, n (%) | 2 (3.3%) | 0 (0%) |
| Gastroesophageal reflux, n (%) | 14 (23.3%) | 2 (14.3%) |
| Hypertension, n (%) | 13 (21.7%) | 5 (35.7%) |
| Diabetes, n (%) | 6 (1%) | 0 (0%) |
| Coronary heart disease, n (%) | 4 (6.7%) | 2 (14.3%) |
| Lung cancer, n (%) | 4 (6.7%) | 0 (0%) |
| ILD score | 8.0 (5.0, 12.5) | 8.5 (8.0, 11.75) |
| Fibrosis feature |  |  |
| Reticulation cysts | 48 (80%) | 12 (85.7%) |
| Thickening of the interlobular septum | 25 (41.7%) | 3 (21.4%) |
| Ground-glass opacity | 17 (28.3%) | 9 (64.2%) |
| Honeycomb cysts | 11 (18.3%) | 3 (21.4%) |
| Traction bronchiectasis | 10 (16.6%) | 1 (7.1%) |
| Emphysema score | 5.0 (3.3, 7.0) | 4.0 (3.0, 6.5) |
| Emphysema feature |  |  |
| Panacinar | 9 (15%) | 2 (14.3%) |
| Centrilobular | 43 (71.7%) | 11 (78.5%) |
| Paraseptal | 8 (13.3%) | 1 (7.1%) |
| CPFE score | 13.0 (10.0, 18.0) | 13.5 (11.0, 21.25) |
| Lung function test parameters^§^ |  |  |
| FEV1% predicted | 67.4 ± 24.3 | 70.5 ± 16.7 |
| FCV1% predicted | 77.8 ± 15.7 | 79.5 ± 13.8 |
| FEV1/FVC (%) | 61.9 ± 17.1 | 79.2 ± 10.5* |
| RV/TLC (%) | 48.9 ± 9.2 | 48.6 ± 8.9 |
| DLCO% predicted | 48.7 ± 18.4 | 37.0 ± 10.1 |
| Laboratory characteristics |  |  |
| ANA, n (%) | 25 (41.7%) | 5 (35.7%) |
| anti-SSA, n (%) | 8 (13.3%) | 3 (21.4%) |
| anti-SSB, n (%) | 8 (13.3%) | 0 (0%) |
| anti-Ro52, n (%) | 7 (11.7%) | 2 (14.2%) |
| RF (IU/mL) | 402 (130, 1047) | 129 (27, 847) |
| anti-CCP(RU/mL) | 199 (63.5, 200) | 67 (28.5, 200) |
| C3 (g/L) | 1.07 ± 0.33 | 1.15 ± 0.30 |
| C4 (g/L) | 0.23 (0.17, 0.31) | 0.28 (0.20, 0.38) |
| IgA (g/L) | 3.47 (2.45, 4.59) | 3.07 (2.62, 3.75) |
| IgG (g/L) | 12.5 (10.5, 15.5) | 13.1 (10.1, 15.2) |
| IgM (g/L) | 1.45 (0.96, 1.89) | 1.52 (0.97, 1.98) |

^§^Lung functional test parameters include 33 patients with RA-CPFE. **P*<0.05, *****P*<0.0001.

**Supplementary table 4. Comparison of smokers and never smokers in patients with RA-CPFE**

|  | **Smokers** | **Never smokers** |
| --- | --- | --- |
| Number | 43 | 31 |
| Sex (male/female) | 42/1 | 18/13**** |
| Age (in years) | 63.5 ± 6.3 | 65.2 ± 9.6 |
| BMI (kg/m^2^) | 20.9 ± 2.9 | 20.8 ± 2.9 |
| Education (≥9 years/＜9 years) | 27/16 | 20/11 |
| Residential location (Urban/ Rural) | 17/26 | 11/20 |
| Duration of RA (in years) | 3.0 (1.0-10.0) | 3.0 (1.0-10.0) |
| Occupational exposure to toxic particles and gas, n (%) | 6 (14%) | 10 (32.3%) |
| Clinical presentations |  |  |
| Joint tenderness, n (%) | 41 (95.3%) | 29 (93.5%) |
| Morning stiffness, n (%) | 32 (74.4%) | 21 (67.7%) |
| Raynaud's phenomenon, n (%) | 6 (14.0%) | 9 (29.0%) |
| Chronic cough, n (%) | 19 (44.2%) | 8 (25.8%) |
| Dyspnea, (n/%) | 21 (48.8%) | 12 (38.7%) |
| Treatment |  |  |
| Steroids | 37 (86%) | 26 (83.9%) |
| Immosuppressor | 43 (100%) | 31 (100%) |
| Home oxygen therapy | 15 (34.9%) | 5 (16.1%) |
| Comorbidities |  |  |
| Allergic diseases, n (%) | 0 (0%) | 2 (6.5%) |
| Gastroesophageal reflux, n (%) | 8 (18.6%) | 8 (25.8%) |
| Hypertension, n (%) | 10 (23.3%) | 8 (25.8%) |
| Diabetes, n (%) | 5 (11.6%) | 1 (3.2%) |
| Coronary heart disease, n (%) | 3 (7.0%) | 3 (9.7%) |
| Lung cancer, n (%) | 3 (7.0%) | 1 (3.2%) |
| ILD score | 8.0 (5.0, 12.0) | 8.0 (7.0, 13.0) |
| Fibrosis feature |  |  |
| Reticulation cysts | 32 (74.4%) | 28 (90.3%) |
| Thickening of the interlobular septum | 20 (46.5%) | 8 (25.8%) |
| Ground-glass opacity | 13 (30.2%) | 13 (41.9%) |
| Honeycomb cysts | 7 (16.3%) | 7 (22.6%) |
| Traction bronchiectasis | 6 (14.0%) | 5 (16.1%) |
| Emphysema score | 5.0 (4.0, 8.0) | 4.0 (3.0, 5.0) ** |
| Emphysema feature |  |  |
| Panacinar | 8 (18.6%) | 3 (9.7%) |
| Centrilobular | 30 (69.8%) | 24 (77.4%) |
| Paraseptal | 5 (11.6%) | 4 (12.9%) |
| CPFE score | 14.0 (10.0, 18.0) | 12.0 (10.0, 18.0) |
| Lung function test parameters^§^ |  |  |
| FEV1% predicted | 65.0 ± 25.3 | 73.8 ± 16.7 |
| FCV1% predicted | 76.7 ± 16.6 | 81.0 ± 11.8 |
| FEV1/FVC (%) | 62.8 ± 17.8 | 69.3 ± 16.3 |
| RV/TLC (%) | 49.0 ± 9.3 | 48.5 ± 9.0 |
| DLCO% predicted | 48.0 ± 16.2 | 44.7 ± 16.2 |
| Laboratory characteristics |  |  |
| ANA, n (%) | 17 (39.5%) | 13 (41.9%) |
| anti-SSA, n (%) | 7 (16.3%) | 4 (12.9%) |
| anti-SSB, n (%) | 2 (4.7%) | 0 (0%) |
| anti-Ro52, n (%) | 5 (11.6%) | 4 (12.9%) |
| RF (IU/mL) | 402 (130, 1047) | 129 (27, 847) |
| anti-CCP(RU/mL) | 199.1 (63.6, 200) | 67.0 (28.5, 200) |
| C3 (g/L) | 1.07 ± 0.33 | 1.15 ± 0.30 |
| C4 (g/L) | 0.23 (0.17, 0.30) | 0.27 (0.19, 0.35) |
| IgA (g/L) | 3.47 (2.45, 4.59) | 3.08 (2.62, 3.76) |
| IgG (g/L) | 12.2 (10.6, 15.5) | 13.1(10.1, 15.2) |
| IgM (g/L) | 1.45 (0.96, 1.89) | 1.52 (0.97, 1.98) |

^§^Lung functional test parameters include 33 patients with RA-CPFE. **P*<0.05, ***P*<0.01, *****P*<0.0001.

**Supplementary table 5. Basic characteristics of the second cohort**

|  | **Healthy Controls**  **(n=36)** | **RA-ILD**  **(n=25)** | **RA-CPFE**  **(n=12)** |
| --- | --- | --- | --- |
| Age (years) | 61.00 ± 9.13 | 60.32 ± 10.38 | 62.25 ± 9.39 |
| Male, n (%) | 12 (33.3) | 7 (28.0) | 9 (75.0) ** |
| BMI (kg/m^2^) | 25.32 ± 3.42 | 22.96 ± 3.30 | 21.23 ± 3.70 |
| Smoker, n (%) | 9 (25.0) | 5 (20.0) | 4 (33.3) |
| Duration of RA (years) | - | 6.0 (3.0, 13.5) | 6.5 (3.0, 13.8) |
| ILD score | - | 7.0 (5.0, 9.0) | 7.0 (5.0, 11.0) |
| Emphysema score | - | - | 3.0 (3.0, 4.0) |
| RF (IU/mL) | - | 78.7 (17.5, 241) | 352 (30, 1070)* |
| anti-CCP(RU/mL) | - | 104.8 (14.0, 194.4) | 51.8 (39.5, 153.5) |
| ANA, n (%) | - | 4 (16.0) | 2 (8.0) |
| Anti-SSA, n (%) | - | 2 (16.6) | 3 (25.0) |

**P*<0.05, ***P*<0.01 (compared to RA-ILD group).

**Supplementary table 6. Correlation between autoantibodies against HBEC and characteristics of RA**

|  | Anti-HBEC IgG | | Anti-HBEC IgM | |
| --- | --- | --- | --- | --- |
|  | Spearman r | *P* values | Spearman r | *P* values |
| Age | 0.06 | n.s. | 0.02 | n.s. |
| RF (IU/ml) | 0.17 | n.s. | 0.50 | 0.0029 |
| anti-CCP（RU/ml) | 0.10 | n.s. | 0.29 | n.s. |
| RA duration （year） | 0.06 | n.s. | -0.11 | n.s. |
| BMI | -0.44 | 0.0067 | -0.40 | 0.0132 |
| ILD score | 0.24 | n.s. | 0.15 | n.s. |
| Emphysema score | -0.03 | n.s. | 0.22 | n.s. |

n.s. not significant.


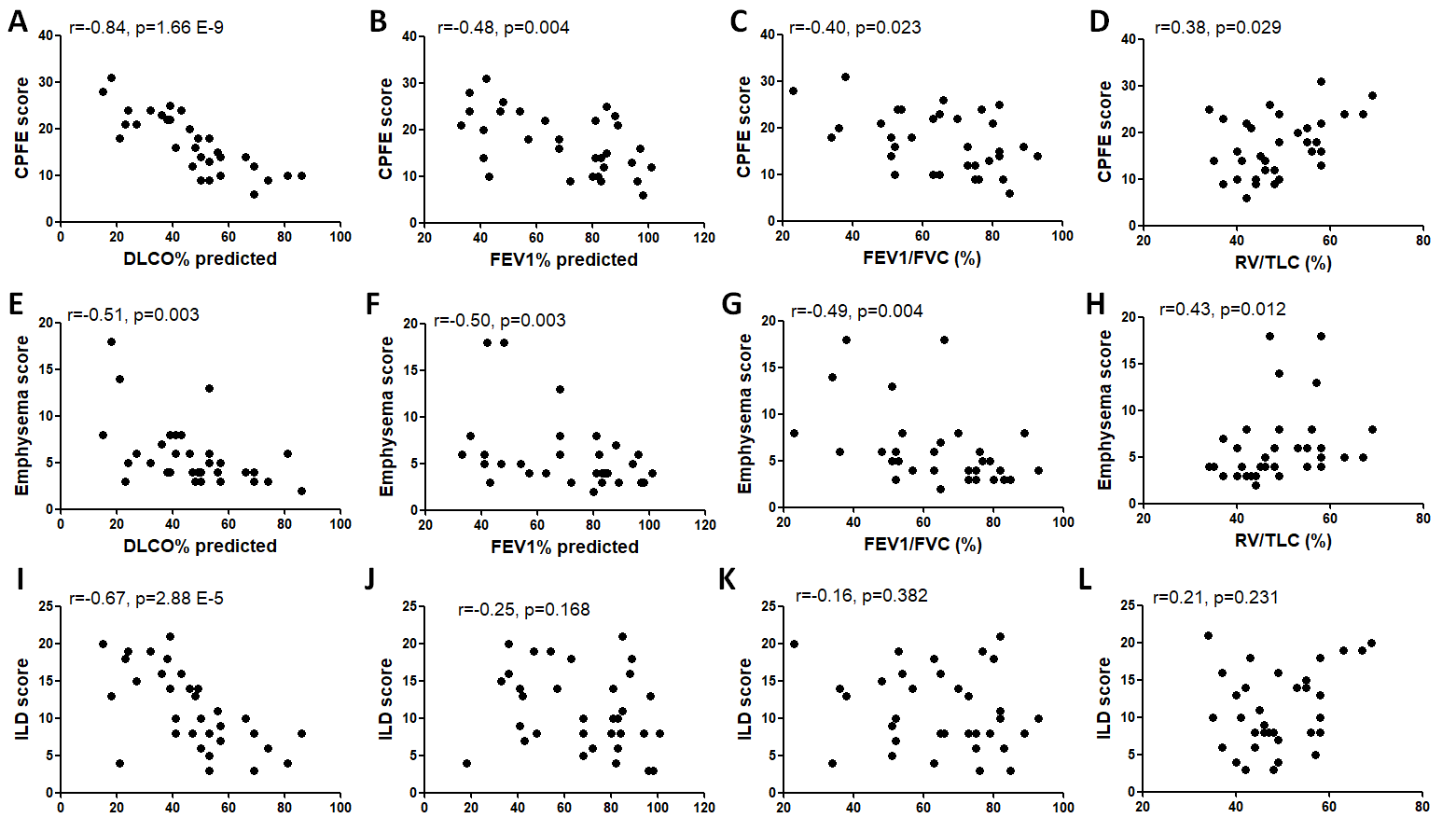


**Supplementary figure 1.** Correlation between disease severity and lung function test in patients with RA-CPFE. Spearmann’s correlation analysis of CPFE score (A-D), emphysema score (E-H), and ILD score (I-L) with DLCO% predicted (A, E, I), FEV1% predicted (B, F, J), FEV1/FVC (%) (C, G, K) and RV/TLC (%) (D, H, L). Correlation coefficient (r) and p values are indicated.
